# Kinesiophobia and its relation to pain characteristics and cognitive affective variables in older adults with chronic pain

**DOI:** 10.1186/s12877-016-0302-6

**Published:** 2016-07-07

**Authors:** Caroline Larsson, Eva Ekvall Hansson, Kristina Sundquist, Ulf Jakobsson

**Affiliations:** Center for Primary Health Care Research, Faculty of Medicine, Clinical Research Centre (CRC), Lund University, Skåne University Hospital, Building 28, floor 11, Jan Waldenströms gata 35, SE-205 02 Malmö, Sweden; Department of Health Science, Lund University, Lund, Sweden; Stanford Prevention Research Center, Stanford University School of Medicine, Stanford, CA USA

**Keywords:** Kinesiophobia, Prevalence, Chronic pain, Older adults

## Abstract

**Background:**

The contribution of kinesiophobia (fear of movement) to the pain experience among older adults has been poorly evaluated. The aim of this study was to study prevalence at baseline, development over a 12-month period and cognitive-affective variables of kinesiophobia in a population-based sample of older adults with chronic pain.

**Methods:**

The study included 433 older adults (+65 years) with chronic pain (mean age 74.8 years) randomly selected using a Swedish register of inhabitants. Kinesiophobia was measured at baseline and 12-month follow-up with the 11-item version of the Tampa Scale of Kinesiophobia (TSK-11). Associations of demographic-, cognitive affective - and pain-related variables to kinesiophobia were analysed with linear regression analyses.

**Results:**

The mean level of kinesiophobia was low. Worsening and recovering from kinesiophobia occurred over time, but the mean level of kinesiophobia remained unchanged (*p* = 0.972). High levels of kinesiophobia (TSK ≥35) were found among frailer and older adults predominately living in care homes, but not dependent on sex. Poor self-perceived health (OR = 8.84) and high pain intensity (OR = 1.22) were significantly associated with kinesiophobia.

**Conclusion:**

Results indicate that potential interventions regarding kinesiophobia among older adults should aim to decrease pain intensity and strengthen health beliefs.

## Background

Kinesiophobia, defined as “an excessive, irrational, and debilitating fear of physical movement and activity resulting from a feeling of vulnerability due to painful injury or reinjury” [1] is found to be a central factor in the process of pain developing from acute to chronic stages [2]. The Cognitive Fear Avoidance Model describes that when a painful experience is interpreted as threatening, it can generate catastrophising cognitions that activity will result in more pain and re/injury. As this goes on, this can lead to avoidance behaviour, which in the long run causes disability, disuse and depression as well as a patient trapped in a cycle of increased fear of pain, more pain and disability [2]. For older adults the consequences of disuse and decreased activity can be serious, increasing the risk for a wide range of health problems, functional decline and premature death [3–5]. Several studies [6, 7], support the validity of the Cognitive Fear Avoidance Model among the elderly [8–10], but there are also some reports of age-related differences. For example, pain-related cognitions might particularly account for age-related differences in the relationship between pain and depression [7, 11]. There is also a lack of generalisability of the fear avoidance model to general populations of older adults. Considering that older adults are especially predisposed to pain as a result of age and that pain among older adults is often of unknown origin [12]. This underlines the need to investigate kinesiophobia in relation to pain in more general populations of older adults too, including the oldest old.

Except for the mechanisms outlined in the fear avoidance model, increasing attention has been given to self-efficacy as a mediating factor between pain-related fear (kinesiophobia) and avoidance behaviour [13]. Self-efficacy relates to the belief in one’s own capacities and individuals with high self-efficacy seem to have a higher ability to manage challenging situations and setbacks than individuals with low self-efficacy [14]. Another cognitive aspect that is strongly associated with the occurrence of pain among older adults is self-perception of health [15]. Poor self-perceived health has also been found to relate to poor recovery from chronic pain [16]. However, the contributions of self-efficacy and health perceptions to the cognitive fear avoidance model among older adults have not been established. Therefore the primary objective of the current study was to explore prevalence at baseline and development of kinesiophobia over a 12-month period in a population-based sample of older adults with chronic pain. In addition, a second aim was to examine the relationships between kinesiophobia, pain characteristics and cognitive affective variables (i.e., self-efficacy, depressed mood and health perception).

## Methods

### Subjects

This study is part of an ongoing prospective population study, in which older adults aged 65 years and older were selected randomly using a Swedish national register of inhabitants (SPAR). At baseline the population study included 2000 older adults who received a questionnaire in the post to which 1141 replied (mean age 74.4 years, range 65–103 years) giving a response rate of 57.8 %. Among those replying to the baseline questionnaire, 433(37.9 %) reported to be suffering from chronic pain (pain duration >3 months) and this group constitutes the sample of the current study (63.5 % women, mean age 74.8, 65–78 years). Subsequent to the baseline questionnaire a follow-up questionnaire was sent to the respondents 12 months later to analyse the development over time.

An analysis of the attrition between those replying at baseline (*n* = 433) and follow up (*n* = 284) indicated that those who were lost at follow-up were slightly older (mean age 78.4 years vs. 74.39 years) but only revealed minor differences for: sex, pain intensity, pain duration.

### Procedure and measurements

All questionnaires were distributed by post together with an accompanying letter explaining the study procedure. It was requested that the questionnaires be returned using enclosed self-addressed, prepaid envelopes. A reminder, letter was sent after 2 weeks.

#### Outcome variable

Kinesiophobia was measured by an 11-item version of the *Tampa Scale of Kinesiophobia* (TSK-11) [1]. The 11 items of the scale each have 4 response options; all anchored with the answers “strongly disagree”, which scores 1 point, and “strongly agree”, which scores 4 points. The total sum score is calculated and can range between 11 and 44 points. A high score indicates strong fear of movement/(re)injury, i.e. high kinesiophobia. TSK-11 has been psychometrically evaluated and has shown good construct validity and reliability among older people (i.e. internal consistency (Cronbach alpha, 0.74–0.87) and test-retest reliability (ICC *r* = 0.747) [17].

#### Demographic variables

Individual demographic variables included sex, age, housing (own home or care homes) and living arrangements (alone or with someone) and marital status (married, never married, widowed or divorced).

#### Pain-related variables

“Chronic pain” was defined as having pain for at least 3 months [18]. Extracted items from the brief screening version of the Multidimensional Pain Inventory (Swedish version) [19] were used to measure intensity, duration, and localisation of pain. “Pain intensity” was measured using the item “Rate the average level of your pain during the last week” responding to a 6-point Likert scale with answers ranging from “No pain at all” (value of 1 point) to “Tremendous amount of pain” (value of 6 points). “Pain duration” was measured in years with pain. “Pain localisation” was measured using a question consisting of 6 answers (upper extremities, lower extremities, shoulder and neck, back and pelvis and other locations). Due to low response rate on the categories “hands” and “feet”, these were combined into the item named “other locations”.

#### Cognitive affective variables

Self-perceived health was measured with a single item “How would you generally describe your health is?” This item was extracted from the 12-item Short-Form Health Survey (SF-12) [20]. Self-perceived health was classified by one of the following responses: excellent health, very good health, good health, fair health and poor health. SF-12 has been found to be valid and reliable in Swedish older adults [21].

Depressed mood was measured with the question; “Have you in the past 3 months been bothered by depressed mood”?, with four response alternatives; “no, not at all”, “yes, little”, “yes rather much” and “yes, very much”. The responses dichotomised into Yes (“yes, little”, “yes, rather much” and “yes, very much”) and No (“No, not at all”). The question about depressed mood originates from a battery of questions about health symptoms previously used among older adults [22, 23].

The General Self-Efficacy scale, GSE, is a generic instrument aiming to measure “optimistic self-beliefs to cope with a variety of difficult demands in life” [24]. The scale consists of 10 items with four answering options. The total sum score is used and ranges between 4–40 points, where a high score indicates high self-efficacy. GSE is commonly used among older people as well as pain patients [25] and has demonstrated good concurrent validity and reliability qualities (internal consistency [alpha: 0.75–0.91], test-retest reliability [*r* = 0, 55–0.67]) [26].

### Ethical considerations

The study was conducted in accordance with the basic ethical principles of medical research according to the Declaration of Helsinki [27] and was approved by the Regional Ethical Review Board in Lund (Reg. no. 2010/683).

### Statistical analysis

Continuous variables are expressed as means and standard deviations while categorical data is presented with ranges and percentiles. Paired sample *t*-test was used in order to compare mean TSK (TSK-11) and the subscales (activity avoidance and somatic focus) between baseline and 12 months follow-up.

To estimate associations for baseline demographic-, cognitive affective - and pain-related variables as a function of TSK-11, simple and multiple linear regression analyses were performed. Variables with *p* -value < 0.05 in the univariate analysis were retained and included in the multiple linear regression analysis. No collinearity problem was detected for any of the models. Analyses were made using PASW 21.0.

## Results

### Demographic descriptions of the sample

In the included sample of older adults with chronic pain (*n* = 433), 275 (63.5 %) were women. The mean duration of pain was 10.2 years and mean pain intensity was 3.2 (SD 1.1). The mean age was 74.8 years (range 65–98) and a majority of the participants were living in their own accommodation (97.4 %) and together with someone (61.7 %) (Table 1).

**Table 1:** Demographic characteristics at baseline in older people with chronic pain^1^ (*n* = 433)

Variable	
Age, mean (SD) range (*n* = 274)	74.8 (7.5) 65–98
Sex, n (%) (*n* = 280)	
Women	275 (63.5)
Living condition, *n* (%) (*n* = 276)	
Own accommodation	413 (97.4)
Care home	11 (2.6)
Living arrangements, *n* (%) (*n* = 276)	
Alone	163 (38.3)
With others	263 (61.7)
Marital status, *n* (%) (*n* = 279)	
Married	235 (54.4)
Single	38 (8.8)
Widow/widowed	95 (21.9)
Divorced	64 (14.8)
Pain duration^2^, mean (SD) range	10.2 (12.2) 0.5–60
Pain intensity^3^, mean (SD) range	3.2 (1.1) 1–6

^1^Pain of duration ≥3 months

^2^Pain duration in years

^3^Pain intensity = “average level of pain in the last week” measured using a 6-point Likert scale with answers ranging from “No pain at all” to “Tremendous amount of pain”

### Kinesiophobia

**Fig. 1:**
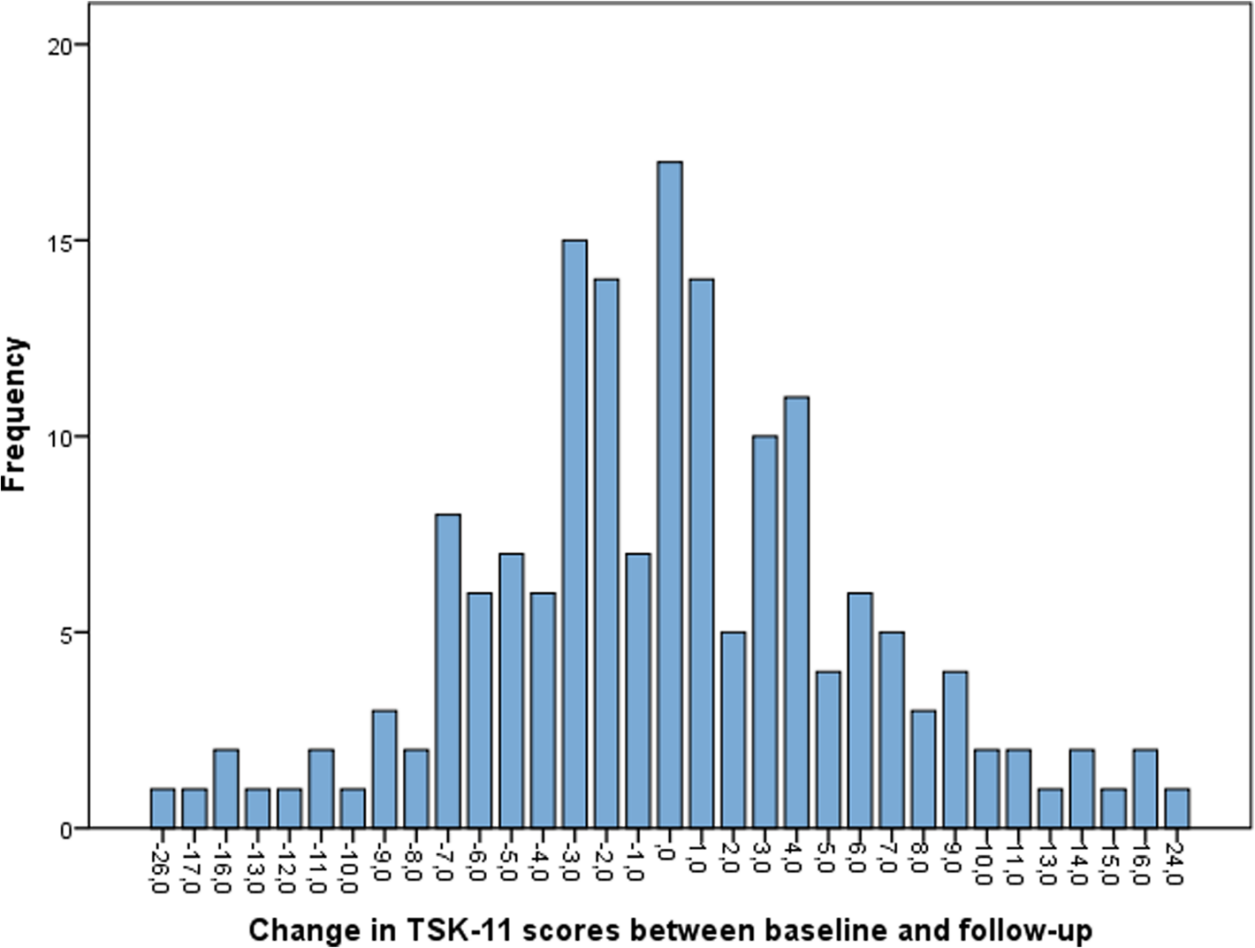
Differences in individual TSK-scores between baseline and follow-up

The mean score of kinesiophobia was 22.8 (SD 8.3), 10 % of the subjects had a score ≥ 35 points. There was no significant change for the mean scores between baseline and follow-up (*p* = 0.97), nor over time for the two sub-scales TSK- activity avoidance (*p* = 0.76) or TSK-somatic focus (*p* = 1.00) (Table 2). However, individual change in kinesiophobia scores (±1) between baselines and follow up was seen among 89.8 % of the subjects and this change ranged between -26 to 24 points (Fig. 1).

**Table 2:** Kinesiophobia at baseline compared to 12 month follow-up in older people with chronic pain^a^

Variable	Baseline	12 month follow-up	*P*-value
Kinesiophobia (TSK-11), mean (SD) range	22.8 (8.3) 11–44	22.7 (8.3) 11–44	0.972^*^
Activity Avoidance (TSK-AA), mean (SD) range	13.4 (5.4) 6–24	13.2 (5.3) 6–24	0.759^*^
Somatic Focus (TSK-SF), mean (SD) range	9.5 (3.7) 5–20	9.6 (3.6) 5–20	1.000^*^

Tampa Scale of Kinesiophobia (TSK-11), sores ranging from 11–44 points with high scores indicating high self-efficacy

^*^Paired *t*-test

^a^Pain of duration ≥3 months

### Variables associated with kinesiophobia at baseline

Age, pain intensity and living arrangements, self-efficacy and “General health-perceived as fair and poor” were all statistically significant associated (*p* < 0.05) with kinesiophobia (Table 3). While the univariate analysis yielded no significant associations between kinesiophobia and the variables sex and pain localisation. Self-efficacy was associated with lower levels of kinesiophobia. When the retained variables were entered into the multiple linear regression analysis as a second step, significant associations with higher levels of kinesiophobia (*p* < 0.05) were found for “pain intensity” (B = 1.22) and “general health” -perceived poor (B = 8.84). However, when comparing the beta values the strongest association was found for pain intensity (β = 0.44) compared to (β = 0.26) for poor health. The adjusted R squared value was 0.18. Among those reporting poor health 43.8 % were living alone, 68.8 % were living in their own accommodation, and a majority (81.1 %) were women and the mean age was 80.1 years (not shown in table). Predictive models were not possible due to the stability of the outcome variable.

**Table 3:** Associations of pain-related and cognitive affective variables on kinesiophobia among older adults with chronic pain^2^. Univariate and multiple linear regression analysis (enter model); for TSK-11^1^

Variables	Univariate linear regression		Multiple linear regression		
	B	*P*-value	B	Beta	*P*-value
Age	0.235	<0. 001	0.111	0.097	0.101
Gender (0 = man, 1 = women)	-0.720	0.466			
Living condition (0 = own accommodation 1 = care home)	10.354	0.005	0.058	0.001	0.988
Pain intensity^3^	2.213	<0.001	1.215	0.439	0.006
Pain localisation					
Other locations ^a^ reference					
Upper extremities	1.267	0. 478			
Lower extremities	2.344	0.133			
Shoulder/neck	0.948	0.068			
Back/pelvis	0.542	0.707			
Depressed mood (0 = no, 1 = yes)	2.991	0.002	0.485	0.029	0.521
General self-efficacy^4^	-0.225	0.002	-0.015	0.012	0.839
Self –perceived health					
Excellent health reference					
Very good health	-0.189	0.945	-2.130	-0.097	0.473
Good health	3.344	0.199	0.800	0.048	0.781
Fair health	7.213	0.006	3.496	0.212	0.230
Poor health	14.492	<0.001	8.838	0.264	0.010

*CI* confidence interval

^1^Tampa Scale of Kinesiophobia (TSK-11), sores ranging from 11–44 points with high scores indicating high Kinesiophobia

^2^Pain of duration ≥3 months

^3^Pain intensity = “average level of pain in the last week” measured using a 6-point Likert scale with answers ranging from “No pain at all” to “Tremendous amount of pain”

^4^General self-efficacy scale (GSE), scores ranging from 10-40 points with high scores indicating high self-efficacy

^a^Other includes: others locations, feet and hands

## Discussion

The main findings of this study were that worsening as well as recovering from kinesiophobia occurred over time, but the mean level of kinesiophobia remained unchanged. Kinesiophobia was significantly associated with pain intensity and poor self-perceived health at baseline. High levels of kinesiophobia were found among frailer and older adults predominately living in care homes, but not dependently of sex.

In this study the mean score of kinesiophobia at baseline was 22.8. It is important to note that kinesiophobia is not a dichotomous characteristic; rather it is expressed as a syndrome which varies in degree [28]. For TSK-11, no cut-off value differentiating between high and low kinesiophobia exists. However, for the 17-item TSK, the total score ranges from17 to 68 and scores > 37 are generally considered as a high level of kinesiophobia [2]. If using the hypothesis that 37 (57 %) on the 68-graded scale represents high kinesiophobia, it would be equal to 35 points on a 44-graded scale. In the current sample, a small proportion (i.e. only the 90th percentile) reached beyond this limit, which in this case would mean that the prevalence of high degrees of kinesiophobia could be considered to be low. Compared to previous studies among younger samples using the 11-item version of TSK scale (where mean values ranged between 25.6 - 36.4) [29–31], lower levels of kinesiophobia were found in this sample representing a general population of older adults with heterogeneous chronic pain. The results correspond to a previous study in a heterogeneous chronic pain sample where a subgroup aged 55–81 years were found to have lower levels of pain-related fear than middle-aged patients [7], indicating that the burden of high kinesiophobia is lower among general populations of older adults compared to younger.

No changes over time were seen when comparing the means of either the subscales (i.e., TSK-AA and TSK-SF) or the total scale. This may indicate that the level of kinesiophobia can be considered relatively constant over time (at least over the period of 1 year) at the group level. However, at the individual level most participants changed their level of kinesiophobia. About 20 % of the participants showed changes greater than one standard deviation. However, what level can be considered clinically relevant has not been established for TSK-11 among older adults with chronic pain. Considering the whole range of the scale (11–44 points), a change of more than 10 % should reasonably be corresponding to a clinically relevant change and equivalent to a change of more than 3 points on TSK-11. Woby et al. [32], suggested in their study that a reduction of 3 points is needed to be 95 % confident that a change has occurred. With regards to our study sample, this suggestion would mean that a clinically relevant change of kinesiophobia occurred among 51.0 % of the participants, and indicating that both worsening (24.6 %) and recovering (26.4 %) from kinesiophobia occurs at old ages.

We also evaluated the relationship between kinesiophobia and possible determinants known to be involved in the pain experience, such as sex, pain intensity, pain localisation depressed mood, self-efficacy and self-perceived health. Apart from sex and pain localisation all variables were individually associated with kinesiophobia in the univariate analysis. Though, for the variable perceived health only the response options “fair” and “poor” health were significant (compared to the reference excellent health). The result of the multiple regression analysis indicated the same result as shown in the univariate analysis but only poor health and pain intensity remained significant. It should be noted that the final model was not completely successful in that it only explained 17 % of the variance, indicating that there might be a risk for multicollinearity, although the tests for multicollinearity did not show this.

The relation between poor health and kinesiophobia is not unexpected given that pain itself may be regarded as a health factor, and found strongly associated with other pain related variables [15], however the result indicates that the contribution of health in the fear avoidance models merits further investigation among elderly populations.

Increasing attention has been given to self-efficacy in explaining pain and pain disability [13, 33]. Woby et al. [13] analysed several cognitive measures in the same model in a sample of chronic low back pain patients. They identified self-efficacy as the strongest predictor of pain disability, suggesting self-efficacy as a mediator between pain-related fear and avoidance behaviours in the fear avoidance model [13]. However, the mediating effect was dependent on the level of self-efficacy, i.e., when self-efficacy was high, elevated pain-related fear did not lead to greater pain and disability. But where self-efficacy was low, elevated pain-related fear was more likely to lead to greater pain and disability. In a recent study among a heterogeneous pain sample a similar mediating effect of self-efficacy was found between pain-related fear and disability but not between pain-related fear and pain severity and depression [34]. In contrast to previous findings, the association of self-efficacy found in the univariate models did not remain significant in the multiple regression analysis in the current study (Table 3). It is possible that a more context-specific measure of pain-related self-efficacy might have a greater impact on kinesiophobia than detected by the General Self Efficacy Scale. For depressed mood, the lack of associations to kinesiophobia is surprising but could be explained by older adults expecting pain and lower activity (i.e. as a part of ageing) and therefore employing different mechanisms for dealing with the pain (i.e. taking medication or ignoring the pain).

### Study limitations

There are some limitations in this study that need to be considered. First, and as always in survey-conducted studies, data rely upon self-reported measures and are thus subjective reports. Interpretation of the result must be done with this in mind. Important to note is that longitudinal research in an older population is unavoidably affected by loss to follow-up. The survey-based data-collection may have increased the risk for selection bias where those most fragile are more likely to not respond. Such systematic attrition may thus undermine the possibility of generalisations. However, an analysis of the attrition between those replying at baseline (*n* = 433) and follow-up (*n* = 284) indicated that those who were lost at follow-up were slightly older (mean age 78.4 years vs. 74.39 years) but only revealed minor differences for sex, pain intensity and pain duration. The details of the attrition analysis have been described previously [35]. Second, no measures of co-morbidity or any specific origin or site of pain were included. Previous studies have shown the applicability of the Cognitive Fear Avoidance Model to vary between different pain types and this may thus have affected the result [36]. However, the current study aimed to explore the kinesiophobia among older adults at population level irrespective of the underlying cause of pain. Moreover, given the fact that only a small change in kinesiophobia was found over a period of 1 year, a longer follow-up period would be merited.

## Conclusion

Low levels of kinesiophobia were found in this heterogeneous sample of older adults with chronic pain. Although both worsening and recovering from kinesiophobia occurred over time, the mean level of kinesiophobia remained unchanged. High levels of kinesiophobia were found among frailer and older adults predominately living in care homes, but not dependent of sex. Poor self-perceived health and high pain intensity were associated in correlation with kinesiophobia and the result of this study will help clinicians in identifying who is most likely to suffer from high kinesiophobia. The results indicate that potential interventions regarding kinesiophobia should aim to decrease pain intensity and strengthen these individuals’ health beliefs.

## Abbreviations

GSE, General Self-Efficacy scale; ICC, Inter class correlation; PASW, Predictive Analytics SoftWare; SD, Standard deviation; SF-12, Short Form Health Survey; SPAR, Swedish personal address register; TSK-11, Tampa Scale of kinesiophobia (11-item version).
